# Cigarette smoke impairs the hematopoietic supportive property of mesenchymal stem cells via the production of reactive oxygen species and NLRP3 activation

**DOI:** 10.1186/s13287-024-03731-2

**Published:** 2024-05-20

**Authors:** Hyun Sung Park, Byung-Chul Lee, Dong-Hoon Chae, Aaron Yu, Jae Han Park, Jiyoung Heo, Myoung Hee Han, Keonwoo Cho, Joong Won Lee, Ji-Won Jung, Cynthia E. Dunbar, Mi-Kyung Oh, Kyung-Rok Yu

**Affiliations:** 1 Department of Agricultural Biotechnology and Research Institute of Agriculture and Life Sciences, Seoul National University, Seoul, 08826 Korea; 2 Department of Biological Sciences, Sookmyung Women’s University, Seoul, Korea; 3 Research Institute of Women’s Health, Sookmyung Women’s University, Seoul, Korea; 4 Division of Allergy and Respiratory Disease Research, Department of Chronic Disease Convergence Research, Korea National Institute of Health, Cheongju, Korea; 5 Translational Stem Cell Biology Branch, National Heart, Lung, and Blood Institute, National Institutes of Health (NIH), Bethesda, MD USA

**Keywords:** Cigarette smoking extract, Mesenchymal stem cells, Reactive oxygen species, NLRP3, Bone marrow niche

## Abstract

**Background:**

Mesenchymal stem cells (MSCs) play important roles in tissue homeostasis by providing a supportive microenvironmental niche for the hematopoietic system. Cigarette smoking induces systemic abnormalities, including an impeded recovery process after hematopoietic stem cell transplantation. However, the role of cigarette smoking-mediated alterations in MSC niche function have not been investigated.

**Methods:**

In the present study, we investigated whether exposure to cigarette smoking extract (CSE) disrupts the hematopoietic niche function of MSCs, and pathways impacted. To investigate the effects on bone marrow (BM)-derived MSCs and support of hematopoietic stem and progenitor cells (HSPCs), mice were repeatedly infused with the CSE named 3R4F, and hematopoietic stem and progenitor cells (HSPCs) supporting function was determined. The impact of 3R4F on MSCs at cellular level were screened by bulk-RNA sequencing and subsequently validated through qRT-PCR. Specific inhibitors were treated to verify the ROS or NLRP3-specific effects, and the cells were then transplanted into the animal model or subjected to coculture with HSPCs.

**Results:**

Both direct ex vivo and systemic in vivo MSC exposure to 3R4F resulted in impaired engraftment in a humanized mouse model. Furthermore, transcriptomic profile analysis showed significantly upregulated signaling pathways related to reactive oxygen species (ROS), inflammation, and aging in 3R4F-treated MSCs. Notably, ingenuity pathway analysis revealed the activation of NLRP3 inflammasome signaling pathway in 3R4F-treated MSCs, and pretreatment with the NLRP3 inhibitor MCC950 rescued the HSPC-supporting ability of 3R4F-treated MSCs.

**Conclusion:**

In conclusion, these findings indicate that exposure to CSE reduces HSPCs supportive function of MSCs by inducing robust ROS production and subsequent NLRP3 activation.

**Supplementary Information:**

The online version contains supplementary material available at 10.1186/s13287-024-03731-2.

## Background

According to the 2021 report from the World Health Organization (WHO), despite decades of knowledge regarding health risks, tobacco continues to account for more than 8 million deaths per year worldwide [1]. Cigarette smoke (CS) contains more than 4,000 toxic chemicals and carcinogens, which contributes to a wide variety of diseases, including cancer, heart disease, lung damage, stroke, and diabetes, resulting in premature death [2, 3]. CS induces cytotoxic DNA damage that leads to tissue damage and inflammation. Among the various target tissues of interest, there is epidemiologic evidence that CS can impact bone marrow function, most notably a strong association with clonal hematopoiesis, with expansion of somatically-mutated clones resulting in a higher risk of hematologic cancers as well as systemic hyperinflammation [4–6]. Murine studies have suggested an impact on both hematopoietic stem and progenitor cells as well as bone marrow (BM) niche elements, including proliferative exhaustion of HSPCs, changes in mesenchymal stromal cells (MSCs), and induction of extramedullary hematopoiesis [4]. CS also has been shown to impact outcomes once HSPC malignant transformation has occurred, with shorter remissions and subsequent survival [7].

In the BM niche microenvironment, mesenchymal stem cells (MSCs) play a pivotal role in regulating self-renewal, survival and differentiation of resident HSPCs through direct contact or paracrine effects [8, 9]. Additionally, MSCs have been developed as a critical tool for maintaining or expanding HSPCs ex vivo prior to transplantation, while preserving their engraftment potential [10, 11]. Cotransplantation of MSCs together with HSPCs facilitates a reduction in the harmful inflammatory responses and contributes to successful engraftment and hematopoietic recovery [12]. However, recent studies suggest that CS can alter multiple characteristics of MSCs, including their ability to support HSPCs [13, 14]. Exposure to CS increases the expression of genes in MSCs that stimulate the proliferation of murine HSPCs in in vitro coculture studies. This exposure potentially results in HSPC exhaustion in vivo, accompanied by impaired engraftment efficiency and a decrease in the absolute number of HSPCs [11]. Nevertheless, there is limited information regarding the pathways impacted by cigarette smoking extract (CSE) on human MSCs and HSPCs.

The production of reactive oxygen species (ROS) by smoking is closely related to the regulation of MSC function. Studies have shown that exposure to ROS derived from CS impairs the regenerative potential of MSCs, reducing their ability to effectively contribute to tissue repair processes [15]. Moreover, it has been reported that increased levels of ROS can negatively impact the function and engraftment capacity of HSPCs [16]. These findings suggest that MSCs exposed to CS have the potential to regulate hematopoietic system via ROS generation. ROS have been identified as factors that trigger apoptosis in multiple immune cells, such as neutrophils and macrophages. In addition, recent studies have suggested that CSE-induced ROS production in human bronchial epithelial cells and monocyte cells activate the nucleotide-binding domain-like receptor protein-3 (NLRP3) inflammasome, leading to pyroptosis, another form of cellular degeneration [17–20]. Although ROS and NLRP3 are associated with apoptosis and pyroptosis in a variety of cell types, the relationship between ROS and NLRP3 in MSCs within the context of hematopoietic niche is not fully understood.

In the present study, we comprehensively investigated the in vitro and in vivo deleterious effects of 3R4F, a reference compound for CSE, on the human HSPC-supportive function of MSCs. We asked whether exposure to CSE could reduce the ability of MSCs to support engraftment of transplanted human HSPCs in a preclinical immunodeficient mouse model. Additionally, we investigated potential pathways involved in bone marrow niche dysfunction, specifically production of reactive oxygen species (ROS) with downstream activation of the NLRP3 inflammasome in MSCs.

## Methods

### Cigarette smoke extract (CSE) preparation

A conventional combustion reference cigarette (3R4F) was obtained from the University of Kentucky (Lexington, KY, USA). Cigarette smoke extract (CSE) was prepared according to ISO/TR 19478-2:2015(en) and Health Canada Method T-115. Briefly, five 3R4F cigarettes were continuously smoked, and CSE were prepared by bubbling smoke into 35 mL of phosphate buffered saline (PBS), which contained approximately 20 µg/mL nicotine. The obtained CSE was diluted as indicated in the study (% v/v).

### Isolation of human CD34^+^ cells from human umbilical cord blood

Human CD34^+^ cells were isolated from human umbilical cord blood as previously described [21]. Briefly, human umbilical cord blood (UCB) was mixed with HetaSep™ solution (STEMCELL Technologies, Vancouver, BC, Canada) at a 5:1 ratio and incubated for 1 h at room temperature. The human mononuclear cells (hMNCs) were isolated from supernatants by density-gradient centrifugation (Ficoll-Paque PLUS, GE Healthcare, Chicago, IL, USA). CD34^+^ hematopoietic stem and progenitor cells (HSPCs) were enriched from hMNCs via magnetic activated cell sorting using human CD34^+^ MicroBead Kit (Miltenyi Biotec, Bergisch Gladbach, Germany) according to the manufacturer’s instructions.

### Xenotransplantation mouse model

NOD-scid-IL2Rγc^−/−^ (NSG) mice were purchased from Jackson Laboratory (Bar Harbor, ME, USA) and housed in a specific pathogen-free facility. Eight-week-old female NSG mice were purchased and housed in a specific pathogen-free facility. For systemic CSE administration, 3R4F (0.5 mL of CSE/kg) was injected intravenously for 4 days, and the control group was injected with 100 µL PBS. To investigate the effect of CSE on the MSC niche, hUCB-derived CD34^+^ HSPCs were cocultured with CSE-treated hMSCs for 3 days and enriched by magnetic activated cell sorting using anti-CD34-conjugated microbeads (Miltenyi Biotec). For NSG bone marrow ablation, busulfan (Sigma‒Aldrich, St. Louis, MO, USA) was dissolved in dimethyl sulfoxide (DMSO, 16 mg/mL) and diluted with PBS at 1:4 ratio. Diluted busulfan was intraperitoneally injected into NSG mice (20 mg/kg). The next day, 5 × 10^4^ CD34^+^ cells were suspended in 100 µL PBS and infused into the NSG mice via intravenous injection.

### Analysis of engraftment

Mouse peripheral blood (mPB) samples were collected from the retro-orbital plexus or tail vein 8 weeks after CD34^+^ HSPC transplantation. At 15 weeks after transplantation, the mice were euthanized by CO_2_, and bone marrow (mBM) and mPB were harvested. mBM was flushed from the femur in RPMI with 10% FBS. Red blood cells were lysed from the PB and BM samples using RBC lysis buffer (Biolegend, San Diego, CA). Single cell suspensions of mPB and mBM were labeled with anti-mouse CD45 (#552848, BD Biosciences), anti-human CD45 (#563879, BD Biosciences), anti-human CD3 (#555341, BD Biosciences), anti-human CD14 (#557831, BD Biosciences), anti-human CD20 (#555623, BD Biosciences), and anti-human CD33 (#564588, BD Biosciences) antibodies and run on an Attune NxT flow cytometer (Thermo Fisher Scientific, Waltham, MA, USA), with analysis by FlowJo V.10 software (BD Biosciences, Franklin Lakes, NJ, USA). To exclude dead cells, 7-AAD (#559925, BD Biosciences) was used.

### Isolation and culture of mouse bone marrow (BM)-MSCs

All animal experiments were approved and conducted in accordance with the Institutional Animal Care and Use Committee (KCDC-029-20-2 A) of the Korea Centers for Disease Control and Prevention. Five-week-old female ICR mice (n = 20) were grouped randomly: Control and 3R4F. Mice in the experimental groups were intravenously injected with 6.25% 3R4F in a total volume of 200 µL PBS, and the control group was injected with 200 µL PBS. All mice received 2 cycles of the injection for 5 consecutive days per week. After euthanasia, mouse BM-MSCs were isolated from the femur and tibia by flushing with DMEM (Gibco, Grand Island, NY, USA). Cells were washed with PBS, and cultured in DMEM supplemented with 10% FBS (Gibco), 1% GlutaMAX (Gibco), 1% antibiotic/antimycotic solution (Gibco), 25 µg/mL EGF (PeproTech), 50 ng/mL bFGF (PeproTech) at 37 °C with 5% CO_2_.

### Ex vivo hematopoietic stem cell and progenitor cell (HSPC) expansion analysis

CD34^+^ HSPCs were enriched from human UCB as mentioned above. Ex vivo HSPC expansion analysis was conducted as previously described [21]. Briefly, mBM-MSCs or hMSCs were treated with 10 µg/mL Mitomycin C for 1 h and seeded at density of 1 × 10^5^ cells per well in a 12-well plate. 1 × 10^4^ of CD34^+^ HSPCs were cocultured with or without mBM-MSCs or hMSCs (MSCs: HSPCs = 10:1). On day 3 of expansion, HSPCs were labeled with human monoclonal antibodies against CD45 (#560178, BD Biosciences), CD34 (#562577, BD Biosciences), and CD90 (#555595, BD Biosciences), and measured with a flow cytometer and analyzed by FlowJo V.10 software (BD Biosciences).

### Human Wharton’s jelly-derived mesenchymal stem cells preparation and culture

The human Wharton’ jelly-derived mesenchymal stem cells (hMSCs) were isolated and cultured as previously described [21]. Briefly, hMSCs were cultured in DMEM (Gibco) supplemented with 10% FBS (Tissue Culture Biologicals), 1% GlutaMAX (Gibco), 1% antibiotic/antimycotic solution (Gibco), 25 ng/mL EGF (PeproTech), and 50 ng/mL bFGF (PeproTech) in a humidified atmosphere containing 5% CO_2_ at 37 °C. Cells were passaged every 3–4 days using 0.05% trypsin/ EDTA. The cells were pretreated with or without N-acetyl cysteine (NAC; 5 mM; Sigma‒Aldrich) or MCC950 (10 µM; Sigma‒Aldrich) before 3R4F treatment.

### RNA isolation and sequencing (RNA-seq)

RNA sequencing (RNA-seq) was performed by Theragen Bio (Seongnam, South Korea) using Illumina technology as previously described, with modifications [22]. Total RNA was extracted and purified from hMSCs incubated with or without 5% 3R4F using TRIzol reagent (Invitrogen, Carlsbad, CA, USA). Libraries were generated using the Illumina TruSeq strand mRNA sample preparation kit (Illumina, San Diego, CA, USA) and sequenced on a NovaSeq 6000 (2 × 150 paired end sequencing, Illumina) according to the manufacturer’s protocol. After removing the adapter sequence and filtering the low-quality reads using an in-house script, the filtered reads were aligned to hg38 using HISAT2. The aligned reads were counted by featureCounts. For differential expression analyses, gene expression for each sample group was quantified with the edgeR R package. Differentially expressed genes (DEGs) in the control and CSE-treated hMSCs were identified based on absolute log2-fold change ≥ 1 and FDR < 0.05. Heatmaps were generated using an in-house script, and clustering analysis was performed using a hierarchical clustering method. Volcano plots of DEGs were generated using ggplot 2 package in R. Data can be found via GEO accession number GSE253105 (https://www.ncbi.nlm.nih.gov/geo/query/acc.cgi?acc=GSE253105).

### Gene Ontology (GO) and gene set enrichment analysis (GSEA)

Differentially expressed genes were subjected to gene enrichment analysis with the R package clusterProfiler, and gene set enrichment analysis (GSEA) was performed using the Broad GSEA application. The significance of the gene sets was calculated using gene set enrichment analysis (GSEA v3.0, https://www.gsea-msigdb.org/gsea/index.jsp). The significance of each factor was calculated using Fisher’s exact test.

### Pathway analysis

Trimmed DEGs (log2-fold change > 2.4, FDR < 0.05) were used for pathway analysis using QIAGEN’s Ingenuity® Pathway Analysis (IPA®, QIAGEN Redwood City, www.qiagen.com/ingenuity).

### Quantitative real-time PCR

Total RNA was extracted using TRIzol reagent (Invitrogen), and 2 µg of total RNA was converted to cDNA using M-MLV Reverse Transcriptase (Promega, Madison, WI, USA). Real-time PCR was performed using 2×SYBR Green Premix (Enzynomics, Daejeon, South Korea) and measured using a CFX96 real-time system (Bio-Rad Laboratories, Hercules, CA). β-actin was used as the reference gene for normalization. The primer sequences for qRT‒PCR are listed in Additional file 2: Table S1.

### Detection of intracellular reactive oxygen species (ROS)

Intracellular reactive oxygen species (ROS) were detected using the peroxide-sensitive fluorophore 2’,7’-dichlorofluorescin diacetate (DCFDA, Sigma‒Aldrich) as previously described [22]. hMSCs were incubated with 5% 3R4F for 72 h. After incubation, the cells were washed with PBS and incubated with 10 µM DCFDA in serum-free culture medium for 30 min at 37 °C. The mean DCFDA fluorescence was analyzed using an Attune NxT flow cytometer (Thermo Fisher Scientific).

### Oxygen consumption rate (OCR) analysis

The oxygen consumption rate (OCR) was measured with the XFe24 Extracellular Flux Analyzer (Seahorse Bioscience, North Billerica, MA, USA). hMSCs were treated with CSE for 72 h and seeded at a density of 4 × 10^4^ cells/well in XFe24 microplates. After 24 h, the growth media were exchanged to Seahorse XF DMEM base medium (Seahorse Bioscience) supplemented with glucose (Sigma‒Aldrich), GlutaMAX (Gibco), and sodium pyruvate (Sigma‒Aldrich). After 30 min of equilibration at 37 °C in a non-CO_2_ incubator, basal OCR was measured according to the manufacturer’s protocol.

### Colony-forming assay

For methylcellulose colony-forming assays, 500 CSE-treated hMSCs were cocultured with CD34^+^ HSPCs. After incubation, HSPCs were mixed with complete MethoCult H4434 complete medium (STEMCELL Technologies) and seeded in 35-mm culture dishes. After 14 days, the number of erythroid burst-forming units (BFU-E), granulocyte-macrophage colony forming units (CFU-GM) and granulocyte/erythrocyte/macrophage/megakaryocyte colony forming units (CFU-GEMM) was determined with manual counting under an inverted light microscope (Olympus Corporation, Tokyo, Japan).

### Western blot analysis

The cells were harvested and lysed using RIPA buffer with protease inhibitor (Thermo Fisher Scientific), and the protein concentrations were determined by the BCA protein assay (Thermo Fisher Scientific). A total of 40 ug of proteins were separated by sodium dodecyl sulfate-polyacrylamide gel electrophoresis (SDS-PAGE) and transferred to nitrocellulose membranes as previously described [23]. Blots were incubated with primary antibodies against NLRP3 (#15101; Cell signaling technology, Danvers, MA, USA), ASC (#67824; Cell signaling technology), pro-IL-1β (sc-32294; 1:1,000 dilution; Santa Cruz Biotechnology, Dallas, TX, USA), cleaved IL-1β (#83186; Cell signaling technology), pro-CASP1 (#3866; Cell signaling technology), cleaved CASP1 (#89332; Cell signaling technology), and GAPDH (sc-32233; Santa Cruz Biotechnology) at the 1:1,000 dilution, followed by an incubation with the secondary antibody conjugated to HRP for 1 h in room temperature. The bands were visualized using SuperSignal West Pico PLUS Chemiluminescent (Thermo Fisher Scientific) and luminescent image analyzer (ChemiDoc XRS + Systems, Bio-Rad Laboratories) at multiple exposure settings. For quantification, ImageJ software (National Institutes of Health) were used to analyzed the bands intensity.

### Statistical analysis

All experiments were performed at least in triplicate. Where data were normally distributed, the significance was determined using one-way ANOVA followed by a Holm‒Sidak multiple comparisons test. The data are presented as the mean ± standard deviation. All statistical analyses were performed using GraphPad Prism 8 (GraphPad Software Inc.; San Diego, CA, USA).

## Results

### 3R4F suppresses BM niche function of MSCs in vivo and ex vivo

**Fig. 1:**
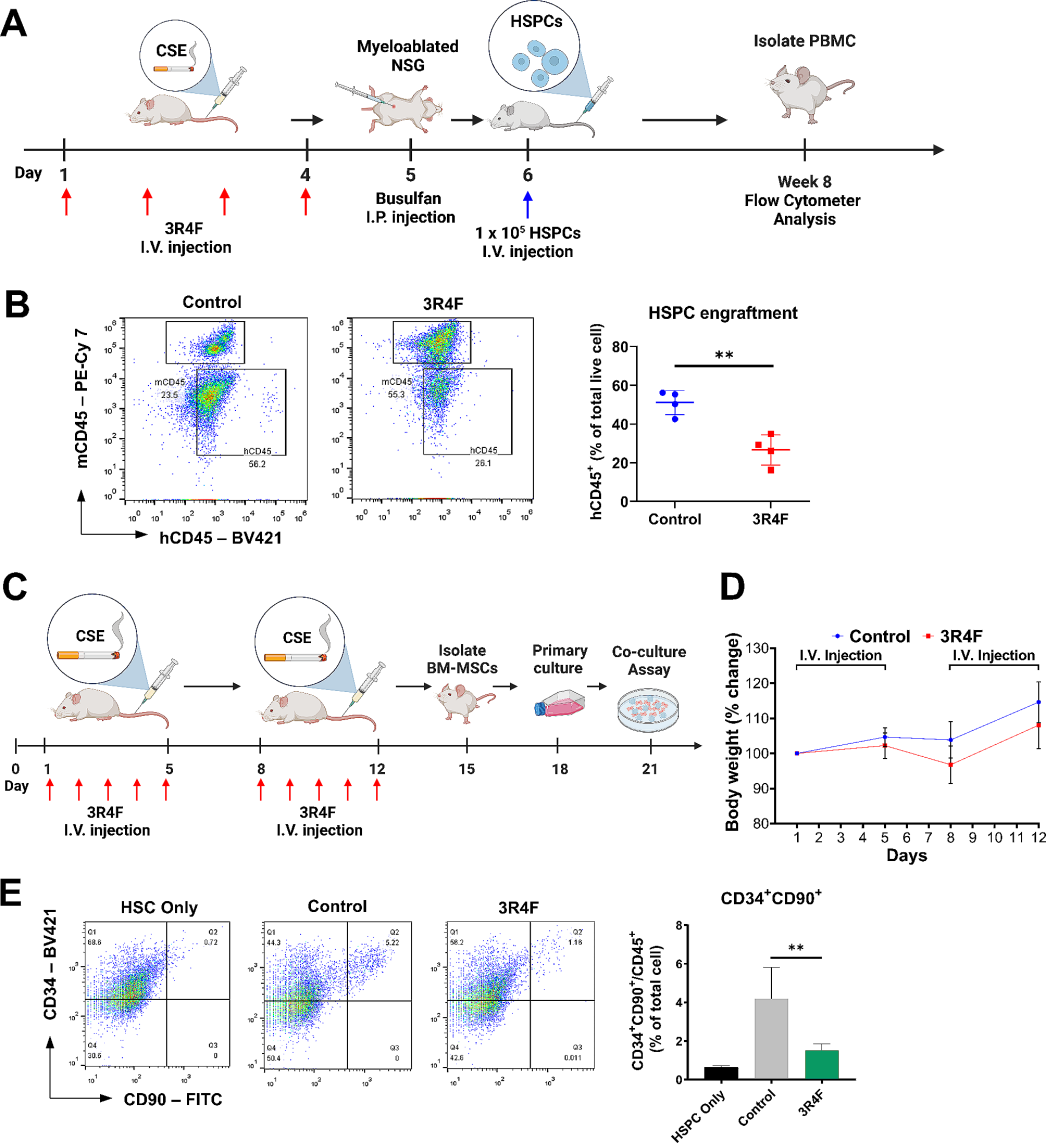
Systemic administration of 3R4F suppresses HSC niche function of mBM-MSCs. (**A**) Schematic outline of systemic 3R4F administration and subsequent CD34^+^ HSPC transplantation in humanized mice. (**B**) Engraftment of human cells was determined from the peripheral blood of 3R4F-treated humanized mice by flow cytometric analysis. Left: representative dot plot images. Right: histogram for repetitive results (n = 4). (**C**) Schematic outline of the isolation of 3R4F-exposed mBM-MSCs and the in vitro BM niche function test. CD34^+^ cells were isolated from hUCB and cocultured with 3R4F-treated mBM-MSCs. (**D**) Body weight change was measured for 12 days. Body weights were normalized using the weight on Day 1 as the denominator (n = 10 mice/group). (**E**) After 3 days of coculture with 3R4F-exposed mBM-MSCs, the CD34^+^CD90^+^ HSPC population in CD45^+^ cells was analyzed using flow cytometry. Left: representative dot plot images. Right: histogram for repetitive results (n = 3). Experiments were performed at least three times. Data are presented as the mean ± S.D. (**p < 0.01)

Previous studies have shown that exposure to cigarette smoke reduces the HSPC pool size [11, 24]. We therefore investigated the effects of CSE on human hematopoiesis using xenotransplantation in NSG mice. Human cord blood-derived CD34^+^ HSPC engraftment as measured by hCD45^+^ % in PB at week 8 post transplantation was significantly reduced in NSG mice given reference cigarette 3R4F extract intravenously (IV) for 4 days prior to transplantation compared to the control group (Fig. 1A, B and Additional file 1: Fig. S1A, B). Next, we examined the impact of more prolonged systemic 3R4F-exposure, administering 3R4F IV 5 days a week for 2 weeks into ICR mice (Fig. 1C, D). On day 15, the mice were sacrificed, and mBM-MSCs were isolated. They were cultured ex vivo with human CD34^+^ HSPCs. After coculture for 3 days, the proportion of the more primitive HSPCs (CD34^+^CD90^+^) was measured. The 3R4F-treated group showed substantial reduction in the percentage of CD34^+^CD90^+^ HSPCs (Fig. 1E). Taken together, systemic exposure to CSE deteriorates the HSPC-supporting ability of mBM-MSCs, resulting in a reduction in HSPC engraftment.

### Transcriptomic profiling reveals that 3R4F exposure elevates inflammation, cellular aging, wound repair, and ROS in hMSCs

We further evaluated the impact of 3R4F on human MSCs by performing in vitro viability and metabolic assays. Direct treatment for 48 h with 1–5% 3R4F did not result in significant cellular death in hMSCs, but 5% 3R4F reduced the metabolic activity (Additional file 1: Fig. S2A, B). Additionally, 5% 3R4F inhibited the migration and increased the adipogenic differentiation of hMSCs (Additional file 1: Fig. S2C, D). Based on these results, 5% 3R4F was used in subsequent experiments.

**Fig. 2:**
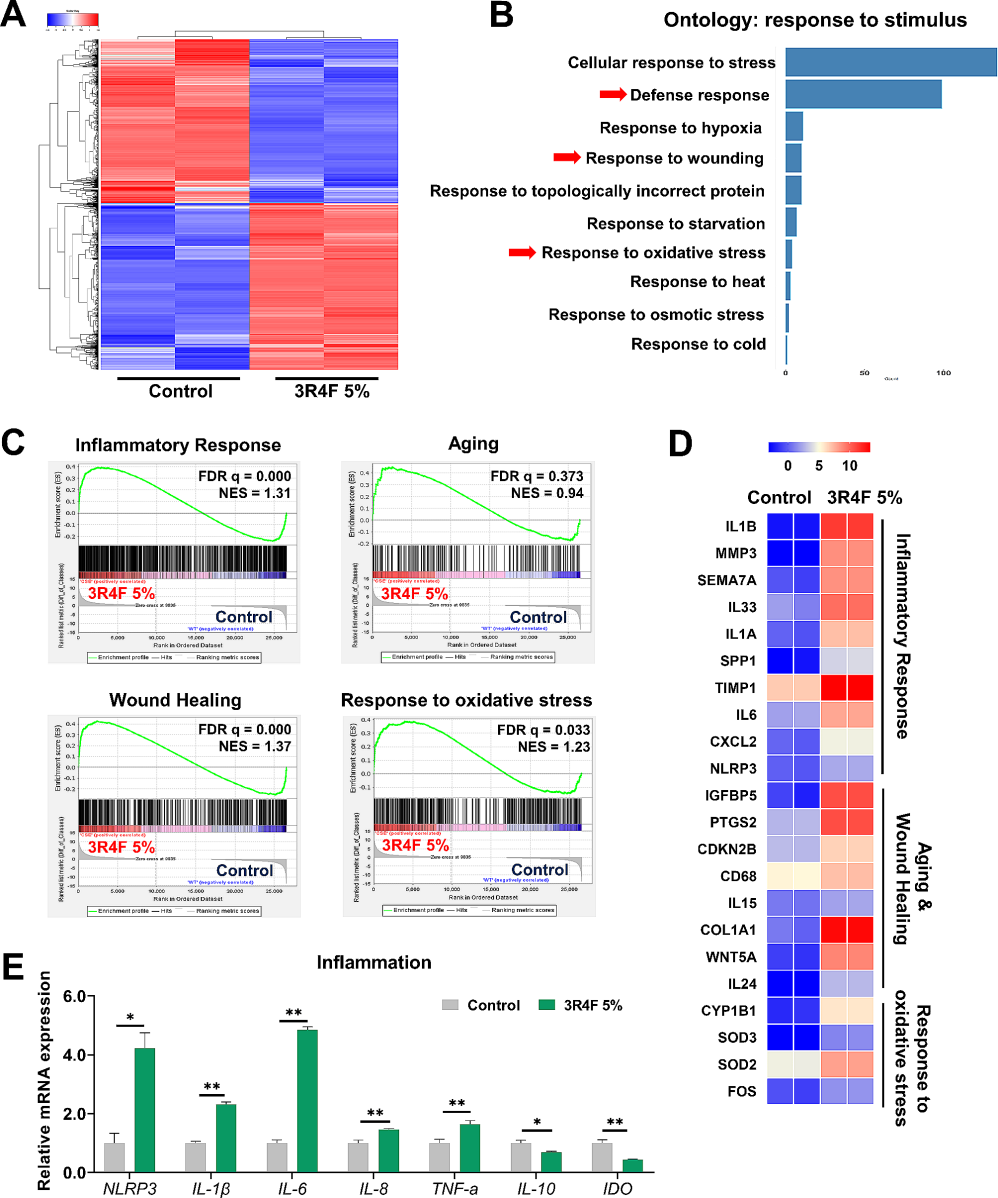
Transcriptomic profiles of 3R4F-treated MSCs present changes toward inflammation, cellular aging, and wound repair. hMSCs were treated with 5% 3R4F for 48 h, and gene expression was analyzed. (**A**) Heatmap of differentially expressed gene (DEG) profiles for the control and the 3R4F-treated groups (FDR < 0.05). The data were clustered hierarchically. (**B**) GO enrichment analysis of DEGs was performed using the Panther biological processes database. (**C**) Enrichment plot of inflammatory response, aging, wound healing, and response to oxidative stress using GSEA GO-BP gene sets. (**D**) Heatmaps of the inflammatory response, aging, wound healing, and response to oxidative stress using GSEA GO-BP gene sets. NES, normalized enrichment score. (**E**) mRNA expression levels of inflammation related genes were analyzed by qRT‒PCR and normalized to β-actin. Data are presented as the mean ± S.D. (*p < 0.05; **p < 0.01)

RNA sequencing was performed to examine the alteration of the transcriptomic profile of 3R4F-treated hMSCs. The differentially expressed genes (DEG) were visualized using hierarchical clustering and a volcano plot (Fig. 2A and Additional file 1: Fig. S3A). Gene Ontology (GO) enrichment analysis identified DEGs that were enriched in the biological processes of defense response, response to wounding, and response to oxidative stress. These processes all fall within the ‘response to stimulus’ upper-category that harbors gene sets related to hMSCs’ physiological responses to CSE (Fig. 2B and Additional file 1: Fig. S3B). Gene set enrichment analysis (GSEA) using the gene ontology biological process (GO BP) gene sets identified four significantly upregulated categories in 3R4F-treated hMSCs: inflammatory response, aging, wound healing, and response to oxidative stress gene sets (Fig. 2C). In inflammatory response, enriched gene sets contained upregulated expression of *NLRP3*, *IL-1β*, and, *IL-6* (Fig. 2D).

To confirm the RNA-seq results, we assessed the expression levels of the proinflammatory genes *NLRP3*, *IL-1β*, *IL-6*, *IL-8*, and *TNF-α* by qRT-PCR. 3R4F treatment increased the expression of these proinflammatory genes in hMSCs. Similarly, we identified that *IL-10* and *IDO*, known immune suppressive factors of MSCs, were both downregulated by 3R4F treatment (Fig. 2E). Additionally, 3R4F inhibited the anti-proliferative effect of hMSCs on T lymphocytes (Additional file 1: Fig. S4A). Collectively, 3R4F perturbs immune homeostasis by altering global gene expression patterns of hMSCs, with functional consequences demonstrated in vitro.

### 3R4F-induced ROS reduce the hematopoietic supportive function of hMSCs

**Fig. 3:**
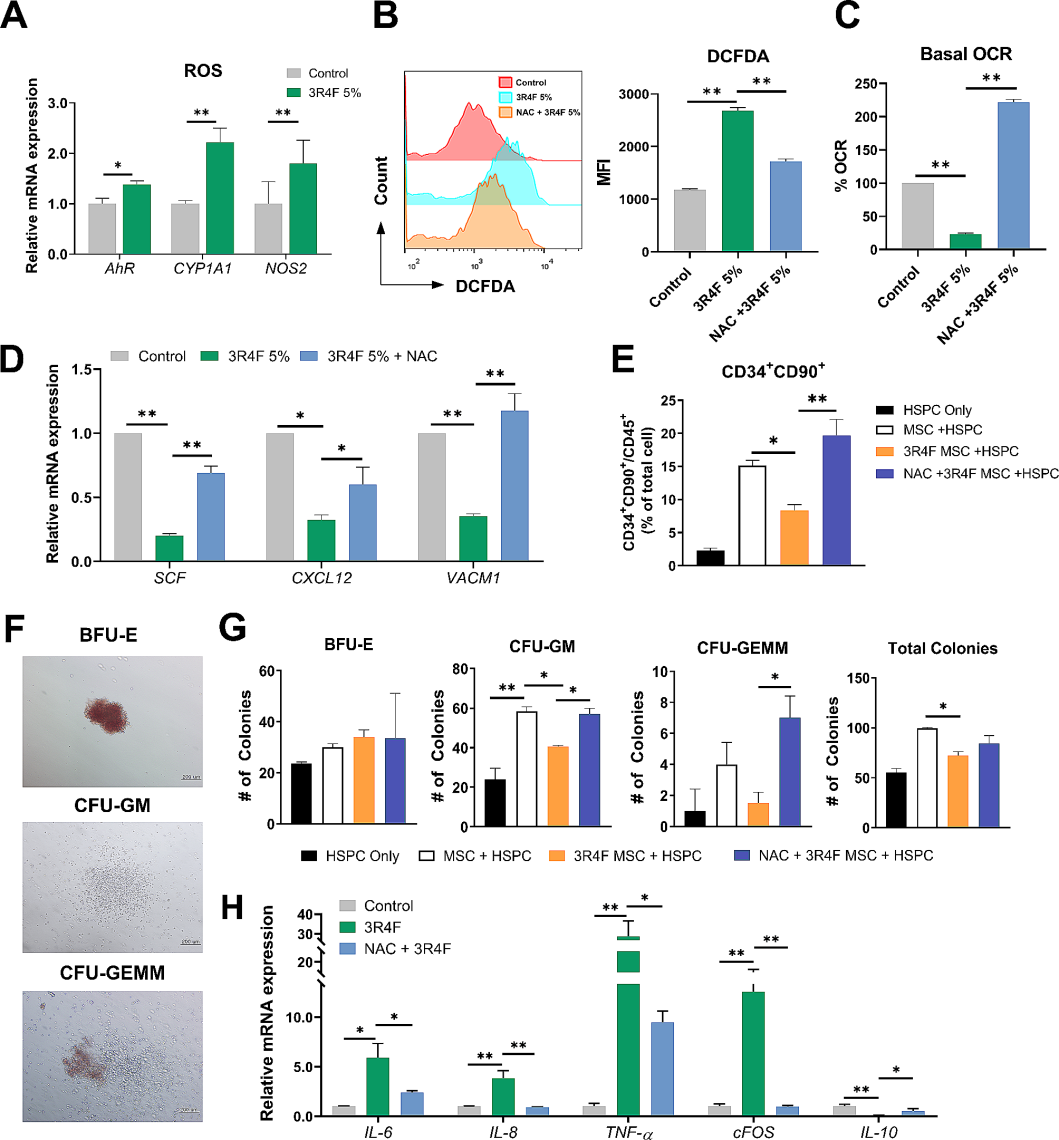
3R4F-exposure induces ROS in hMSCs, resulting in a reduction in the supporting ability of HSPCs. (**A**) mRNA expression levels of ROS-related genes (*AhR*, *CYP1A1*, and *NOS2*) in 3R4F-treated hMSCs were analyzed by qRT‒PCR. The expression level of each gene was normalized to that of β-actin. (**B-D**) hMSCs were pretreated with or without NAC for 1 h and followed by treatment with 5% 3R4F for 72 h. (**B**) Intracellular ROS levels were measured by flow cytometric analysis by using the ROS-sensitive fluorophore 2’,7’-dichlorofluorescin diacetate (DCFDA). (**C**) The basal level of the cellular respiration rate (oxygen consumption rate [OCR]) was determined by using an XFe24 Extracellular Flux Analyzer. (**D**) mRNA expression level of HSPC niche related genes in 3R4F-treated MSCs were analyzed by qRT-PCR and normalized to that of β-actin. (**E-G**) hCD34^+^ HSPCs were cocultured with 3R4F-treated hMSCs with or without NAC pretreatment for 72 h. (**E**) After 3 days of coculture, the CD34^+^CD90^+^ HSPC population in CD45^+^ cells were analyzed using flow cytometry. (**F, G**) After coculture, HSPCs were seeded in methylcellulose colony formation medium and cultured for 2 weeks. (**F**) Representative colony morphologies (Scale bar: 200 μm). (**G**) The number of BFU-E, CFU-GM, CFU-GEMM, and the total sum of all colonies were quantified. (**H**) hMSCs were pretreated with or without NAC for 1 h and followed by treatment with 5% 3R4F for 48 h. mRNA expression levels of inflammation-related genes (*IL-6*, *IL-8*, *TNF-α*, *cFOS*, and *IL-10*) were determined by qRT-PCR. The expression level of each gene was normalized to that of β-actin. The data are presented as the mean ± S.D. of three independent experiments (*p < 0.05; **p < 0.01). BFU-E – Burst forming Erythrocyte; GM – Granulocyte/Macrophage; GEMM – Granulocyte/Erythrocyte/Macrophage/Megakaryocyte.

It has been reported that accumulated ROS inhibit the HSPC-supporting ability of stromal cells in aged mice [25]. In a similar context, our RNA-seq results indicated that 3R4F-treated hMSCs developed a gene expression GO profile suggesting excess ROS (Fig. 2C, D). We measured the mRNA expression level of ROS-related genes including *AhR, CYP1A1*, and *NOS2*. All were upregulated in hMSCs following 3R4F exposure (Fig. 3A). We also verified that exposure led to the induction of intracellular ROS by the DCFDA assay. 3R4F-treated hMSCs showed increased intracellular ROS levels, which were reduced by pretreatment with N-acetyl cysteine (NAC; ROS inhibitor) (Fig. 3B). A recent study showed that oxidative stress by ROS inhibits the metabolic activity of hMSCs [26]. We performed real-time metabolic analysis to measure the basal respiration of hMSCs with 5% 3R4F exposure, showing significantly reduced basal oxygen consumption rate (OCR). Pretreatment with NAC attenuated the reduced OCR of the 3R4F-treated hMSCs (Fig. 3C).

In vitro coculture of human HSPCs on MSCs can preserve or augment their viability, self-renewal and engraftment capabilities [27, 28]. To assess the impact of CSE on this supportive function of hMSCs on HSPCs, we assessed the mRNA expression levels of the niche-related genes *SCF, CXCL12*, and *VCAM1* in hMSCs. Their expression was suppressed by exposure to 3R4F and recovered by the introduction of a ROS inhibitor (Fig. 3D). Next, human CD34^+^ HPSCs were cocultured with hMSCs with or without 3R4F exposure to investigate the effect of CSE on the supportive role of MSCs in maintaining the more primitive fraction of CD34^+^CD90^+^ cells as quantified by flow cytometric analysis. The proportion of CD34^+^CD90^+^ HSPCs was improved by the presence of hMSCs, and this supportive ability was decreased by 3R4F pretreatment of the hMSCs, while ROS inhibition by NAC during hMSC exposure to 3R4F rescued the supportive effect of the hMSCs on CD34^+^CD90^+^ cells (Fig. 3E and Additional file 1: Fig. S4B). Plating of hematopoietic colonies (CFU) following coculture of CD34^+^ HSPC on hMSCs pre-treated with 3R4F showed a reduction in myeloid lineage colonies (CFU-GM and CFU-GEMM), again rescued by ROS inhibition during hMSC 3R4F pre-treatment. There was no significant change in the number of erythroid colonies (Fig. 3F, G and Additional file 1: Fig. S4C). Given that a proinflammatory milieu has been reported to disrupt HSPC-supporting properties of hMSCs [29], we measured inflammation-related gene expression in 3R4F-treated hMSCs. Proinflammatory genes, including *IL-6, IL-8, TNF-α*, and *cFOS*, were increased, whereas *IL-10*, an anti-inflammatory gene, was reduced. Pretreatment with NAC protected against these changes in gene expression (Fig. 3H).

### NAC ameliorates the 3R4F-induced dysfunction of the HSPC-supporting ability of hMSCs

**Fig. 4:**
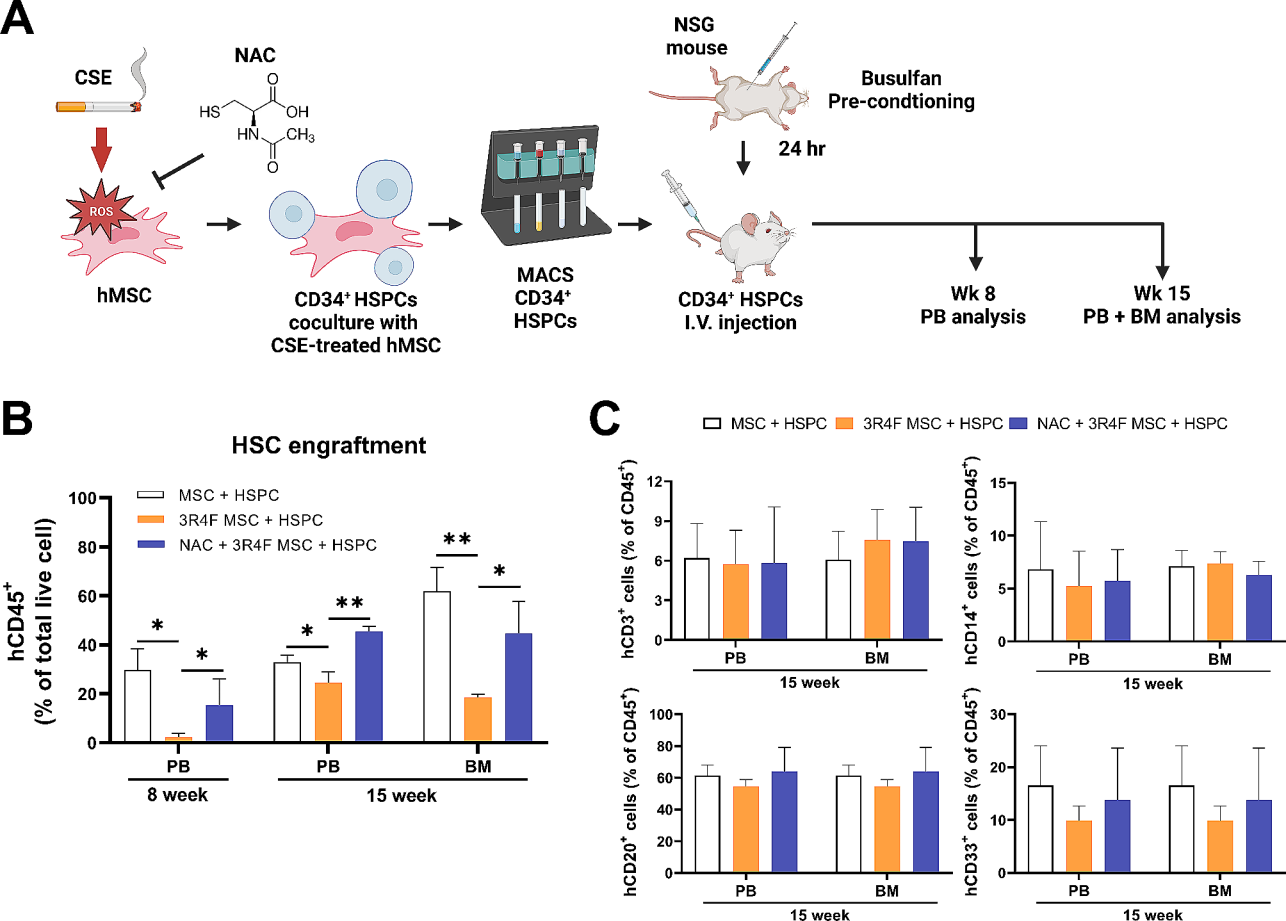
ROS inhibition restores the impaired HSPC supporting ability of hMSCs and engraftment in xenotransplantation model. (**A**) A schematic diagram of ROS inhibition in hMSCs and following hMSC-cocultured HSPC transplantation. hMSCs were pretreated with or without NAC for 1 h, and followed by treatment with 5% 3R4F for 72 h. CD34^+^ HSPC were cocultured with 3R4F-treated hMSC. After 3 days, cocultured HSPCs were harvested, enriched via magnetic sorting, and intravenously injected into NSG mice. PB and BM samples were collected at the indicated timepoints for further analyses. (**B**) HSPC engraftment was evaluated by determining hCD45^+^ cells from PB 8 weeks post transplantation. (**C**) Repopulation of blood lineage cells (hCD3^+^ T cells, hCD14^+^ monocytes, hCD20^+^ B cells, hCD33^+^ myeloid cells) was analyzed in the PB and BM of NSG mice at 15 weeks post transplantation. n = 3–4 mice/group. The data are presented as the mean ± S.D. of three independent experiments (*p < 0.05; **p < 0.01)

We next studied the impact of CSE on the ability of hMSCs to support the engraftment potential of hHSPCs. Human CD34^+^ HSPCs were cocultured for 3 days with control or 3R4F-treated hMSCs, with or without NAC, and administered to NSG mice (Fig. 4A). Blood was collected 8 weeks after transplantation and analyzed by flow cytometry for hCD45 to measure human HSPC engraftment. Coculture with 3R4F-treated hMSCs markedly decreased the engraftment capacity of HSPCs compared to coculture with control hMSCs. Moreover, this reduction was attenuated by pretreatment with NAC (Fig. 4B). At 15 weeks post transplantation, BM and PB samples were harvested and the fraction of hCD45^+^ cells was analyzed. Similar to the 8 weeks PB analysis, both BM and PB from 15 weeks showed a significantly reduced hCD45^+^ proportion for mice receiving HSPCs cultured with 3R4F treated hMSC that was restored by ROS inhibition (Fig. 4B). Lineage analysis within the human CD45^+^ compartment showed no difference in HSPC production of myeloid, T cell and B cell lineages when exposed to 3R4F-treated hMSCs compared to control hMSCs (Fig. 4C). Collectively, these results suggest that 3R4F-induced ROS disrupt the niche function of hMSCs in the engraftment capacity of HSPCs during in vitro culture, while ROS inhibition effectively restores the decreased engraftment.

### 3R4F-induced ROS regulates the hematopoietic supportive function via NLRP3 in hMSCs

**Fig. 5:**
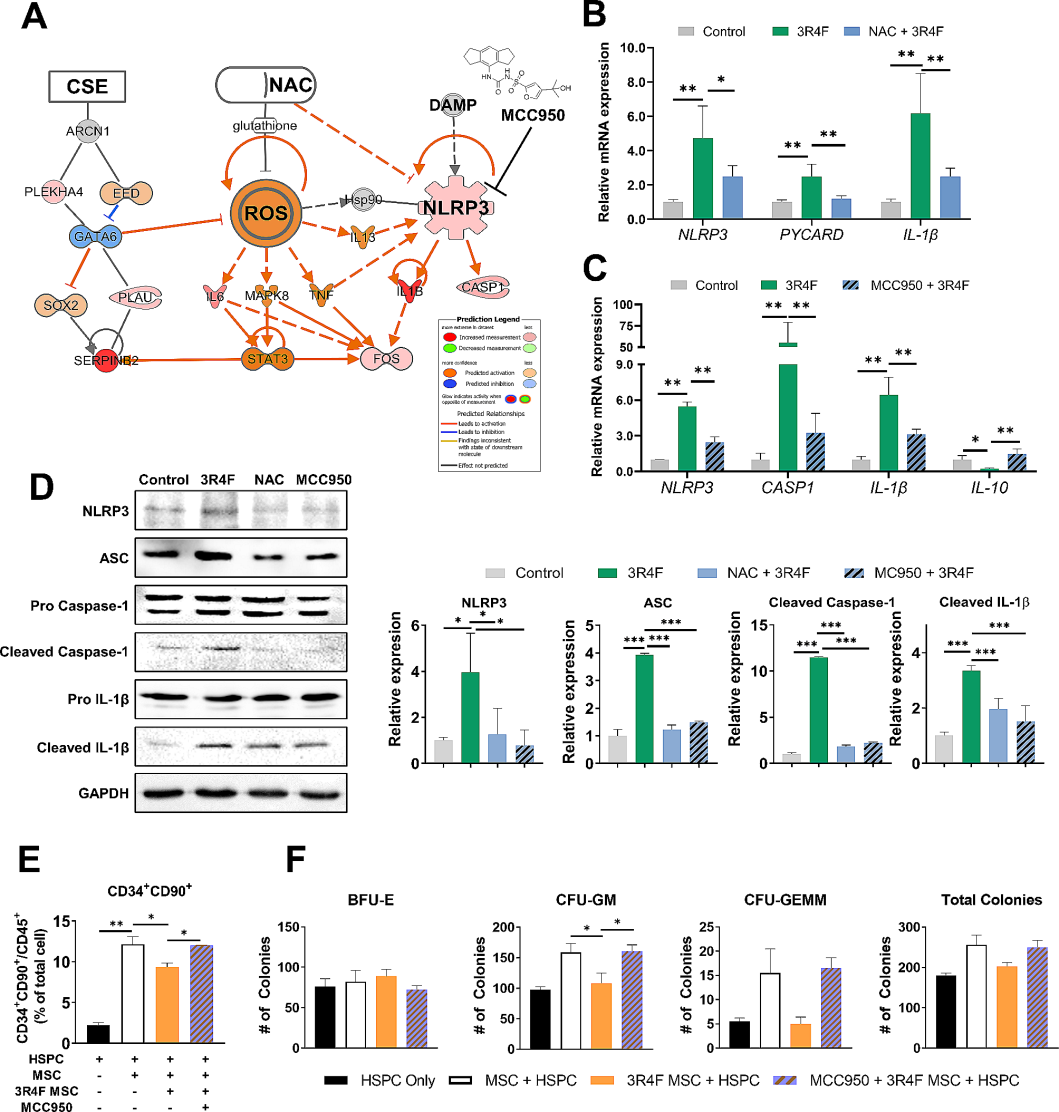
3R4F-induced ROS impair hematopoiesis supportive ability of hMSCs via the NLRP3 inflammasome. (**A**) An illustration of 3R4F-induced ROS activation and the NLRP3 expression-related pathway was depicted based on the DEG profile of hMSCs. IPA software was used for *in silico* analysis. (**B, C, D**) hMSCs were pretreated with or without NAC or MCC950 for 1 h and followed by treatment with 5% 3R4F for 72 h. (**B**) The mRNA expression levels of NLRP3 inflammasome related genes (*NLRP3*, *PYCARD*, and *IL-1β*) was analyzed by qRT‒PCR. The expression level of each gene was normalized to that of β-actin. (**C**) mRNA expression level of inflammation related genes (*NLRP3*, *CASP1, IL-1β*, and *IL-10*) were analyzed by qRT‒PCR. The expression level of each gene was normalized to that of β-actin. (**D**) The expression of NLRP3, ASC, pro/cleaved Caspase-1, and pro/cleaved IL-1β was analyzed and quantified by western blotting. (**E**) MCC950-treated hMSCs were cocultured with hCD34^+^ HSPCs for 3days. CD34^+^CD90^+^ HSPC population in CD45^+^ cells was analyzed using flow cytometry. (**F**) Three days after coculture, HSPCs were seeded in methylcellulose colony formation medium and cultured for 2 weeks. The number of BFU-E, CFU-GM, CFU-GEMM, and the total sum of all colonies were quantified. Data are presented as the mean ± S.D. (*p < 0.05; **p < 0.01; ***p < 0.001). BFU-E – Burst forming Erythrocyte; GM – Granulocyte/Macrophage; GEMM – Granulocyte/Erythrocyte/Macrophage/Megakaryocyte.

Given the above results, we hypothesized that CSE impacted the ability of hMSCs to support HSPCs via activation of the NLRP3 inflammasome. We observed that ROS and NLRP3 were positively regulated by each other in the analysis of the DEG profile using IPA (Fig. 5A). qRT-PCR analysis confirmed that ROS scavenging by NAC reduced the expression of NLRP3 inflammasome-related genes in 3R4F-treated hMSCs (Fig. 5B). We examined the impact of adding MCC950, an NLRP3 inhibitor, during hMSC 3R4F pre-treatment, and assessed its effects on HSPC-supporting abilities. We confirmed that the 3R4F-induced NLRP3 inflammasome pathway was partially blocked by MCC950, with decreases in the expression of *NLRP3, IL-1β*, and *CASP1* (Fig. 5C). Consistent with the mRNA expression results, the NLRP3 inflammasome-related proteins (NLRP3, ASC, CASP1, and IL-1β) expression levels were induced in hMSCs by the 3R4F treatment, while pretreatment with NAC and MCC950 efficiently prevented the effect of 3R4F (Fig. 5D). Pretreatment with MCC950 indeed ameliorated the impact of 3R4F exposure on hMSCs in supporting CD34 + CD90 + HSPCs (Fig. 5E), and on the number of CFU-GM and CFU-GEMM colonies (Fig. 5F and Additional file 1: Fig. S5A). Thus, inhibition of NLRP3 restores the HSPC-supporting ability of CSE-treated hMSCs.

## Discussion

Although it has been observed that smokers tend to exhibit reduced posttransplant survival rates and increased mortality following allogeneic HSPC transplantation [30–33], the potential role of impaired hematopoietic niche function has not been explored in depth. We conducted a comprehensive analysis of the influence of CSE on MSC characteristics and MSC-HSPC interactions. The reference compound we utilized, 3R4F, contains 9.4 mg of tar and 0.73 mg of nicotine per cigarette [34, 35] and is commonly used for toxicity assessments, such ROS production, inflammation, and cellular damage to a range of cells, including bronchial epithelial cells and vascular endothelial cells [35–37]. Past studies have suggested that CS impairs the characteristics of BM-MSCs in the bone marrow environment. However, these studies have primarily focused on phenotypic changes in the bone marrow microenvironment, such as reductions in HSPC pool size or decreased proliferative capacity of BM-MSCs [11, 38].

CSE-induced excessive production of ROS and resultant oxidative stress are widely recognized as key contributors to the development of diverse physiological impairments and disorders. We carried out an in-depth investigation of the impact of CSE on BM niche function, both in vivo via exposure of mice to 3R4F prior to transplantation of HSPCs, and ex vivo via exposure of hMSCs to 3R4F prior to coculture with HSPCs. Pre-treatment of mice resulted in impaired engraftment of HSPCs, potentially due to impact on the BM niche. Meanwhile, we analyzed the transcriptomic profile of CSE-exposed hMSCs to elucidate the underlying mechanisms and revealed the activation of pathways related to tissue damage, inflammation, and aging, consistent with previously documented effects. Notably, an increase in the production of ROS was observed. Through *in silico* and functional analyses, we explored the potential mechanisms through which elevated ROS levels may compromise the HSPC-supportive role of MSCs. The remarkable decrease in HSPC engraftment efficiency resulting from exposure to 3R4F in the NSG mice model implies that these effects might be further exacerbated within a biological system, notably in humans. Furthermore, we provide evidence that inhibition of ROS restores CSE-mediated HSPC-supporting ability of hMSCs, suggesting that CSE-induced ROS may play a key role in the interaction between BM niche stem cells and HSPC engraftment.

Previous studies have shown that the NLRP3 inflammasome is involved in the development of hematologic diseases and plays a role in regulating hematopoiesis. S100A9 activates the ROS-dependent NLRP3 inflammasome and induces pyroptotic cell death and clonal expansion of HSPCs in myelodysplastic syndrome (MDS) patients [39, 40]. However, there is limited information regarding the relationship between ROS-induced activation of the NLRP3 inflammasome and MSCs within the hematopoietic niche. Our studies demonstrated that the NLRP3 inflammasome was activated, followed by ROS induction, and the suppression of ROS or NLRP3 activation possibly restored the impaired HSPC-supporting ability of MSCs. This study thus demonstrates that the activation of ROS/NLRP3 inflammasome serves as a causative factor in impaired HSC engraftment postsmoking exposure, potentially impeding the natural hematopoietic supportive function of hMSCs.

It is suggested that stem cell properties derived from different sources may be distinct depending on their tissue of origin. Consequently, tailoring the utilization of MSCs or MSC-derived extracellular vesicles (EVs) based on their sources and specialized function is recommended [41–43]. Therefore, subsequent research will potentially envision more specific phenotyping and mechanistic studies of MSCs from different origins focusing on their direct HSPC-supporting capacity. Nevertheless, it is noteworthy that the direct assessment of the impact of human MSCs in an in vivo setting, achieved by transplanting human HSPCs cocultured with human MSCs to NSG mice, carries scientific and preclinical significance.

In light of these considerations, it is evident that in future endeavors involving HSPC transplantation or cell-based therapeutic approaches, the influence of smoking when employing MSCs should be underscored and carefully considered. Consequently, this study systematically analyzed the effects that smoking can have on transplanted HSPCs and hematopoietic niche and, furthermore, has provided clinical significance by emphasizing the need for the selective application of MSCs as adjuncts in HSPC transplantation [8, 44, 45].

## Conclusions

In conclusion, our study elucidates that activation of the ROS/NLRP3 pathway disrupts the hematopoietic support function of hMSCs following CSE treatment, thereby impairing HSPC engraftment. We found that tissue damage, inflammation, and aging-related genes were significantly upregulated upon CSE exposure. Notably, inhibition of CSE-induced ROS/NLRP3 pathway was effective in restoring the HSPCs supportive function of CSE-treated hMSCs. Taken together, our research provides valuable insights into the niche signals that influence hematopoietic system regulation in the bone marrow. This, in turn, contributes potential strategies to mitigate the adverse effects of smoking on stem cell therapies.

### Electronic supplementary material

Below is the link to the electronic supplementary material.

Supplementary Material 1

Supplementary Material 2

## Data Availability

All data are included in the text and supplementary materials. Data details are available from the corresponding author on request.

## Abbreviations

MSC: Mesenchymal stem cell
CSE: Cigarette smoking extract
BM: Bone marrow
HSPC: Hematopoietic stem and progenitor cells
ROS: Reactive oxygen species
OCR: oxygen consumption rate
CFU: Colony forming unit
NAC: N-acetyl-l-cysteine
NLRP3: Nucleotide-binding domain-like receptor protein-3
UCB: Umbilical cord blood
MNC: Mononuclear cells
DEG: Differentially expressed genes
GOBP: Gene ontology biological process
