# Supplementary material for: Cigarette smoke impairs the hematopoietic supportive property of mesenchymal stem cells via the production of reactive oxygen species and NLRP3 activation
Source: Stem Cell Res Ther. 2024 May 20;15:145. doi: 10.1186/s13287-024-03731-2 (PMC11103961; doi:10.1186/s13287-024-03731-2)
Supplement: Supplementary file 1 — Supplementary Material 1 [file 13287_2024_3731_MOESM1_ESM.docx]

**Supplementary Information**

**Apoptosis assay**

To determine the apoptosis of CSE-treated hMSCs, an annexin V/7AAD staining assay was performed as previously described (1). hMSCs were seeded into a 24-well plate with and without various concentrations of 3R4F (0 ~ 5%) for 48 hr. Then, the cells were stained with annexin V (#556419, BD Biosciences, Franklin Lakes, NJ, USA) and 7-AAD (BD Biosciences) following the manufacturer’s instructions, and then analyzed using FlowJo V.10 software (#559925, BD Biosciences).

**MTT assay**

To analyze the impact of CSE on the metabolic activity of hMSCs, 3-(4,5-dimethylthiazol-2-yl)-2,5-diphenyl tetrazolium bromide (MTT; Sigma‒Aldrich, St. Louis, MO, USA) assay was performed as previously described (2). hMSCs were seeded at a density of 5 x 10^4^ in 24-well plate with 3R4F (0 ~ 5%) for 48 hr. MTT solution (0.5 mg/ mL, Sigma‒Aldrich) was added to each well and incubated for 3 hr at 37°C. The formazan precipitate was solubilized with the 0.04 M HCl in isopropanol, and the solution was transferred to a 96-well microplate to measure absorbance at 570 nm using a Synergy™ H1 microplate reader (Biotek, Winooski, WT, USA).

**Scratch wound healing assay**

hMSCs were seeded at a density of 1.5 x 10^5^ cells in a 24-well plate and cultured overnight to attain 100% of the final density of the confluent monolayer. Subsequently, the cells were incubated with 10 µg/mL mitomycin C (MMC, Sigma‒Aldrich) for 1 hr to inhibit further cell growth. A sterile pipette tip was used to create a scratch in the cellular layer, after which the cells were either treated with 5% 3R4F or left untreated. Wound closure within the scratched area was observed after 18 hr, and the distances were calculated using ImageJ software (National Institutes of Health, Bethesda, MD, USA).

***In vitro* differentiation assay**

The differentiation capacity of hMSCs was determined as previously described (1). In brief, hMSCs were plated in 12-well plate at a density of 5 x 10^4^ cells with or without 5% 3R4F for 72 hr. For adipogenic and osteogenic differentiation, cells at 70-80% confluence were incubated with StemMACS™ Adipodiff Media and OsteoDiff Media (Miltenyi Biotec) with or without the 5% 3R4F. The induction media were changed every 3 days. After 21 days, calcium deposition in osteogenic cells were stained with Alizarin Red S (Sigma‒Aldrich) and subsequently dissolved in 100 mM cetylpyridinium chloride (Sigma‒Aldrich) for quantification. For adipogenesis, lipid droplets were visualized by staining with Oil Red O (Abcam, Cambridge, UK). The Oil Red O stained cells were dissolved in 100 % isopropanol and the solution’s absorbance was measured at 490 nm using a Synergy™ H1 microplate reader (Biotek).

**Human peripheral blood mononuclear cells (PBMCs) isolation**

Human peripheral bloods from healthy donors were provided from the Korean Red Cross Blood Services (Seoul, Korea) under approval of the Institutional Review Board (IRB) of the Seoul National University (IRB No. E2210/002-007). Human peripheral blood mononuclear cells (hPBMCs) were isolated by centrifugation over a Ficoll-Hypaque density gradient as previously described (2), and resuspended in RPMI 1640 medium supplemented with 10% FBS.

**T cell proliferation assay**

To assess the impact of 3R4F-induced proinflammatory cytokines on the immunomodulation effect of hMSCs, T cells proliferation assay was conducted as previously described (1). Initially, hMSCs were treated with 10 μg/ml MMC for 1 hr and then seeded at density of 1 x 10^4^ cells per well in a 96-well plate. Subsequently, hPBMCs were labeled with 2 μM 5,6-carboxyfluorescein succinimidyl ester (CFSE; Thermo Fisher Scientific, Waltham, MA, USA), and 1 x 10^5^ hPBMCs were added to the MMC-treated hMSCs wells, along with anti-CD3/CD28 microbeads (Gibco) and recombinant human IL-2 (30 U/mL, PeproTech) to induce T cell proliferation. After 6 days, T cell proliferation was determined by flow cytometry using CD45 (#552848, BD Biosciences), CD3 (#563109, BD Biosciences), CD4 (#555349, BD Biosciences), and CD8 (#562428, BD Biosciences). Viable subpopulation of T cells was selected by gating 7AAD negative cells.


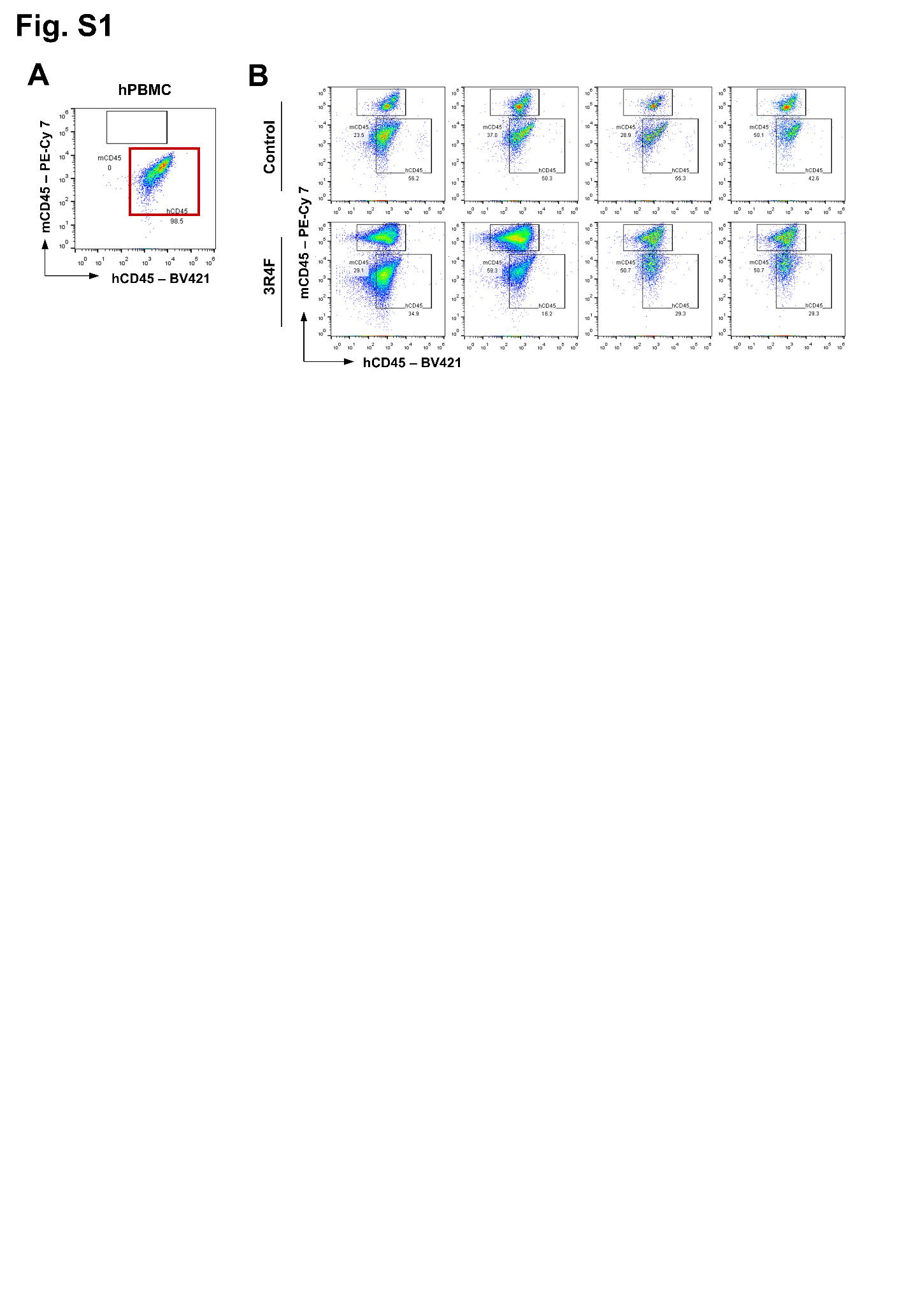


**Supplementary Figure 1. Systemic administration of 3R4F suppresses HSPC engraftment in NSG mice. (A)** Dot plot for CD45^+^ hPBMC only. **(B)** Engraftment of human cells were determined from the peripheral blood of 3R4F treated humanized mice by flow cytometric analysis. The dot plot for all HSPC engrafted NSG mouse from the control group and the 3R4F group (*n* = 4).

**
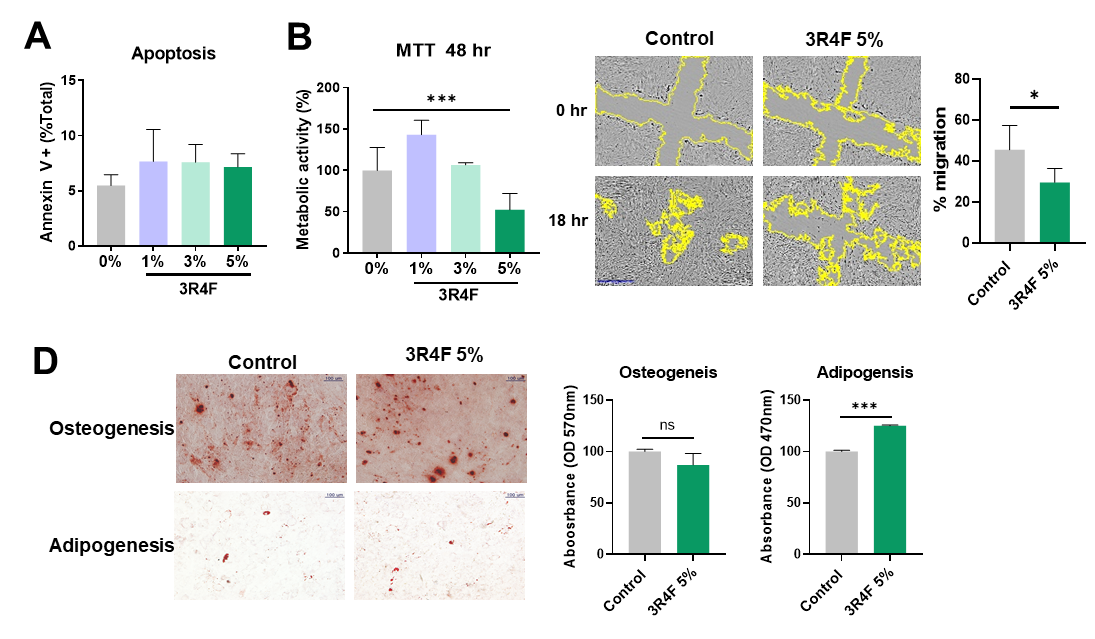
**
**Supplementary Figure 2. Exposure to 3R4F hinders the regenerative potential of hMSCs.** **(A)** hMSCs were treated with different concentration of 3R4F. After 48 hr, hMSCs were labeled with Annexin-V FITC and 7-AAD for flow cytometry analysis. **(B)** Metabolic activity of hMSCs treated with different concentrations of 3R4F were assessed after 48 hr by MTT assay. **(C)** A wound healing assay was performed with a confluent monolayer of hMSCs. After the scratch, the cells were treated with various concentration of 5% 3R4F. Migration into scratched area was measured after 18 hr. (Scale bar: 200 μm). **(D**) 5% 3R4F-treated hMSCs were cultured with osteogenic and adipogenic differentiation medium for 21 days. Representative light microscopic images of osteocytes and adipocytes. Calcium deposits (Alizarin Red S) and lipid droplets (Oil Red O) were obtained (scale bar: 100 μm). Absorbance measurements using a spectrophotometer were used for quantification. The data are presented as the mean ± S.D. of three independent experiments (*p<0.05; **p<0.01).

**
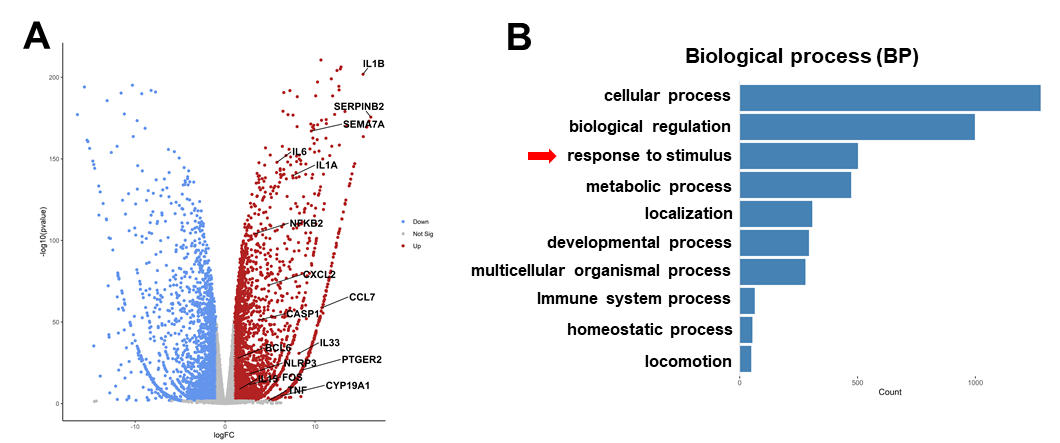
**

**Supplementary Figure 3. Transcriptomic profiles of 3R4F treated MSCs presents changes toward inflammation, cellular aging, and wound repair. (A)** A volcano plot highlighting Differentially Expressed Genes (DEGs) in control and 3R4F-treated hMSCs, focusing on genes related to inflammation, cellular aging, and wound repair. **(B)** A histogram representing the biological processes in the Gene Ontology (GO) classification for DEGs in both control and 3R4F-treated hMSCs.


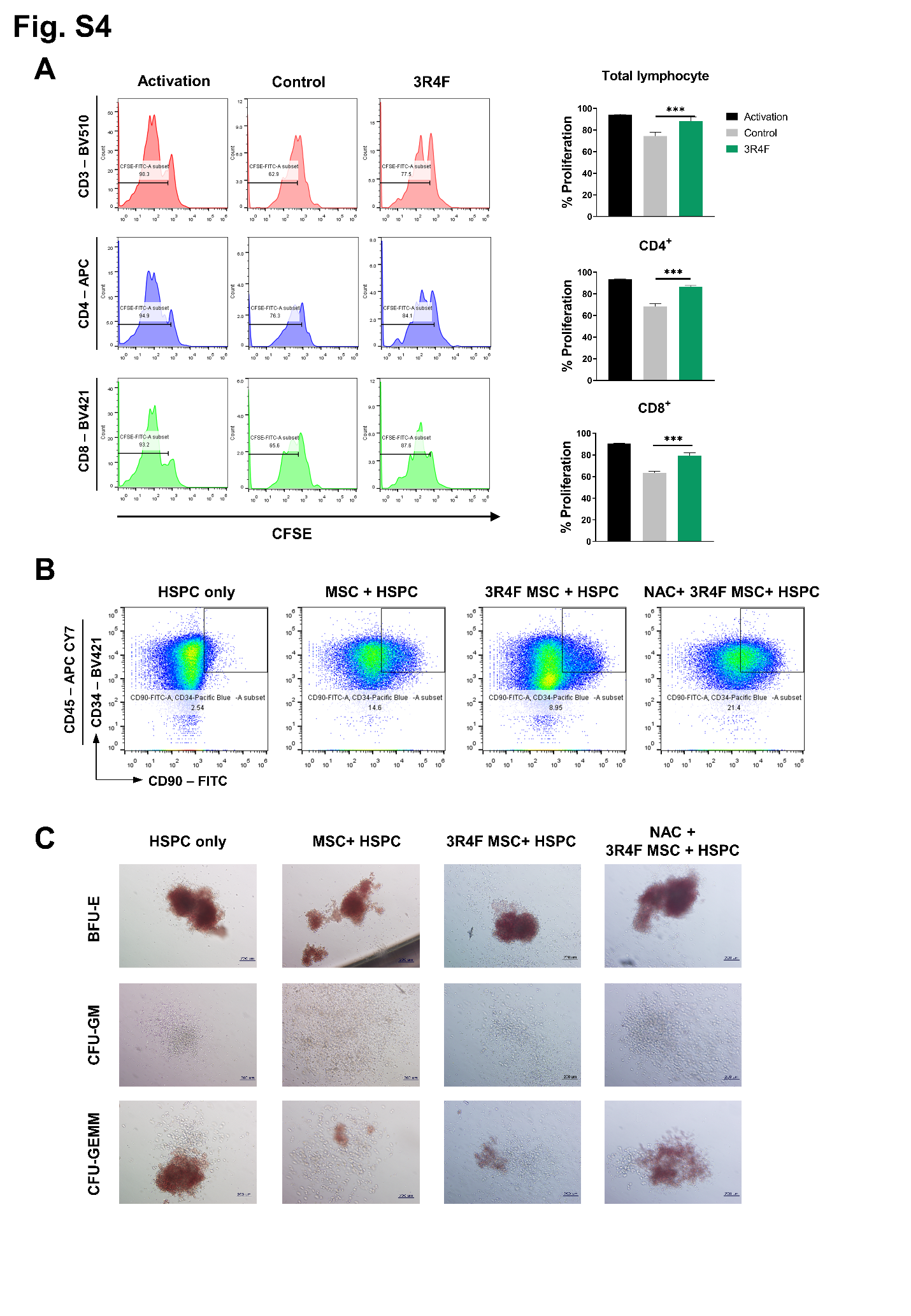


**Supplementary Figure 4. 3R4F inhibits the immunomodulatory effects and hematopoietic supportive function of hMSCs.** **(A)** hMSCs were mitotically inactivated after treating 5% 3R4F for 72 hr. Cells were cocultured with CFSE-labeled hPBMCs that were activated by CD3/CD28 Dynabeads and IL-2. After three days of coculture, the percentage of T cell proliferation was measured using flow cytometry. The data are presented as the mean ± S.D. of three independent experiments (*p<0.05; **p<0.01-). **(B)** hMSC were pretreated with or without NAC for 1 hr and followed by treatment with 5% 3R4F for 72 hr. Pretreated hMSCs were cocultured with hCD34^+^ HSPCs for 3days. Representative dot plot of CD34^+^CD90^+^ HSPC population in CD45^+^cells after 3 days of coculture. **(C)** Representative colony morphologies from each group (Scale bar: 200 μm). BFU-E – Burst forming Erythrocyte; GM – Granulocyte/Macrophage; GEMM – Granulocyte/Erythrocyte/Macrophage/Megakaryocyte.





**Supplementary Figure 5. MCC950 ameliorates the 3R4F inhibitory effect on the hMSCs’ hematopoietic supportive functions.** **(A)** Representative colony morphologies from each group (Scale bar: 200 μm). BFU-E – Burst forming Erythrocyte; GM – Granulocyte/Macrophage; GEMM – Granulocyte/Erythrocyte/Macrophage/Megakaryocyte.

**References**

1. Park HS, Oh MK, Lee JW, Chae DH, Joo H, Kang JY, et al. Diesel Exhaust Particles Impair Therapeutic Effect of Human Wharton's Jelly-Derived Mesenchymal Stem Cells against Experimental Colitis through ROS/ERK/cFos Signaling Pathway. Int J Stem Cells. 2022;15(2):203-16.

2. Joo H, Oh M-K, Kang JY, Park HS, Chae D-H, Kim J, et al. Extracellular Vesicles from Thapsigargin-Treated Mesenchymal Stem Cells Ameliorated Experimental Colitis via Enhanced Immunomodulatory Properties. Biomedicines. 2021;9(2):209.
