# Supplementary material for: Cigarette smoke impairs the hematopoietic supportive property of mesenchymal stem cells via the production of reactive oxygen species and NLRP3 activation
Source: Stem Cell Res Ther. 2024 May 20;15:145. doi: 10.1186/s13287-024-03731-2 (PMC11103961; doi:10.1186/s13287-024-03731-2)
Supplement: Supplementary file 2 — Supplementary Material 2 [file 13287_2024_3731_MOESM2_ESM.docx]

**Table** **S1. Primers sequences list**

| **Gene** | **Forward Primers (5'-3')** | **Reverse Primers (5'-3')** |
| --- | --- | --- |
| *β-actin* | CACCATTGGCAATGAGCGGTTC | AGGTCTTTGCGGATGTCCACGT |
| *NLRP3* | CTATCCTCCCCAGAAGGGCT | TATCTGAACCCCACTTCGGC |
| *PYCARD* | AGCTCACCGCTAACGTGCTGC | CTTGGCTGCCGACTGAGGAG |
| *IL-1β* | CCACAGACCTTCCAGGAGAATG | GTGCAGTTCAGTGATCGTACAGG |
| *IL-6* | CTCCTTCTCCACAAGCGCC | GATGCCGTCGAGGATGTACC |
| *IL-8* | GAGAGTGATTGAGAGTGGACCAC | CACAACCCTCTGCACCCAGTTT |
| *IL-10* | GGCACCCAGTCTGAGAACAG | GGCAACCCAGGTAACCCTTA |
| *IDO* | GCCTGATCTCATAGAGTCTGGC | TGCATCCCAGAACTAGACGTGC |
| *TNF-α* | CAGAGGGAAGAGTTCCCCAG | CCTTGGTCTGGTAGGAGACG |
| *Ahr* | GTCGTCTAAGGTGTCTGCTGGA | CGCAAACAAAGCCAACTGAGGTG |
| *CYP1A1* | GATTGAGCACTGTCAGGAGAAGC | ATGAGGCTCCAGGAGATAGCAG |
| *SCF* | CCCTGAAGACTCGGGCTA | CAATTAACGCAAATGAGAGCC |
| *CXCL12* | TTG TAG CCC GGC TGA AGA ACA | GGCAGGCCCTTCCCTAACAC |
| *VCAM1* | GACCTGTTCCAGCGAGGGTCTA | CTTCCATCCTCATAGCAATTAAGGTG |
| *CASP1* | GCTGAGGTTGACATCACAGGCA | TGC TGTCAGAGGTCTTGTGCTC |

List of primers used for RT-qPCR.
